# C-terminal kinesin motor KIFC1 participates in facilitating proper cell division of human seminoma

**DOI:** 10.18632/oncotarget.18139

**Published:** 2017-05-24

**Authors:** Yu-Xi Xiao, Hao-Qing Shen, Zhen-Yu She, Li Sheng, Qian-Qian Chen, Yu-Lan Chu, Fu-Qing Tan, Wan-Xi Yang

**Affiliations:** ^1^ The Sperm Laboratory, College of Life Sciences, Zhejiang University, Hangzhou, China; ^2^ The First Affiliated Hospital, College of Medicine, Zhejiang University, Hangzhou, China

**Keywords:** KIFC1, kinesin-14, seminoma, testis cancer, cell division

## Abstract

C-terminus kinesin motor KIFC1 is known for centrosome clustering in cancer cells with supernumerary centrosomes. KIFC1 crosslinks and glides on microtubules (MT) to assist normal bipolar spindle formation to avoid multi-polar cell division, which might be fatal. Testis cancer is the most common human cancer among young men. However, the gene expression profiles of testis cancer is still not complete and the expression of the C-terminus kinesin motor KIFC1 in testis cancer has not yet been examined. We found that KIFC1 is enriched in seminoma tissues in both mRNA level and protein level, and is specifically enriched in the cells that divide actively. Cell experiments showed that KIFC1 may be essential in cell division, but not essential in metastasis. Based on subcellular immuno-florescent staining results, we also described the localization of KIFC1 during cell cycle. By expressing ΔC-FLAG peptide in the cells, we found that the tail domain of KIFC1 might be essential for the dynamic disassociation of KIFC1, and the motor domain of KIFC1 might be essential for the degradation of KIFC1. Our work provides a new perspective for seminoma research.

## INTRODUCTION

Testis cancer is the most common cancer type among young men, and seminoma is one of the most common testis cancers in the world. Unfortunately, however, the incidence rates of testis cancers continue to increase, while most of the other cancers are reported to tend to be stable or declining from 2003 to 2013 [1]. And in United States alone, there are up to 8,720 new cases estimated in 2016 [1]. Moreover, gene expression profiles of testis cancers are not yet well established.

Human kinesin-14 family member proteins, whose motor domain is on C-terminus, moves along the MT towards the minus ends instead of the plus ends as other kinesins do [2], include KIFC1, KIFC2 and KIFC3. It has been reported that human KIFC3 is highly expressed in human docetaxel resistant breast cancers, and increases the amount of free tubulins in human breast cancer. So KIFC3 is thought to contribute to docetaxel resistance [3]. KIFC1 is known for its centrosome clustering in multi-centrosome cells, organizing multi-polar spindles into pseudo-bipolar spindles to avoid multi-polar division [4]. Therefore, KIFC1 is considered to be a new-generation chemotherapy target for cancer, as it plays important roles in cancer cell division, which usually possess more than two centrosomes, but is not as important in normal cells [5–7]. Meanwhile, KIFC1 is also found to be enriched in numerous of cancer types [8–14], but there is no report on testis cancers. Hence, three different small molecule inhibitors specifically targeting KIFC1 have been designed [15–17]. As KIFC1 is known for handling with supernumerary centrosomes, it is also thought to be linked with cell migration [18] and chromosome instability [19]. Other functions of KIFC1 in cancer cells including driving tumor malignancy by interacting with cyclins [20] and inducing drug resistance against taxane [21] and tamoxifane [22] in breast [9] and/or prostate cancer [21]. KIFC1 is also observed to be linked with metastasis in NSCL (non-small cell lung cancer) [12] and triple-negative breast cancer [23]. Besides, KIFC1 is also found functional in normal cell types. It is found to be essential in vesicular and organelle trafficking [24–27], spermiogenesis [28], oocyte development [29] and embryo gestation [30].

There are several explanations for the up-regulation of KIFC1 in breast cancers. *kifc1* gene is on chromosome 6, and is found amplified relative to the number of chromosome 6 centromere [20]. Specifically in estrogen-receptor positive breast tumors cells, the up-regulation of KIFC1 is also related to a transcription factor p110 CUX1 [31, 32]. KIFC1 might also be decreased with the repression of bromodomain protein ANCCA (AAA nuclear coregulator cancer associated) [22].

Here we show that KIFC1 is also enriched in human seminoma tissues and might facilitate the proper division of seminoma cell division. We also show that the function of KIFC1 is more than centrosome-clustering and MT organization. KIFC1 might also be involved in lining up the chromosomes, trafficking of organelles during cell cycle, and the length of the anaphase. We also show that the tail domain of KIFC1 might be essential for both the dynamic disassociation of KIFC1, and the ubiquitination site of KIFC1 might be on the motor domain.

## RESULTS

### KIFC1 is significantly enriched in human seminoma tissue samples

**Figure 1:**
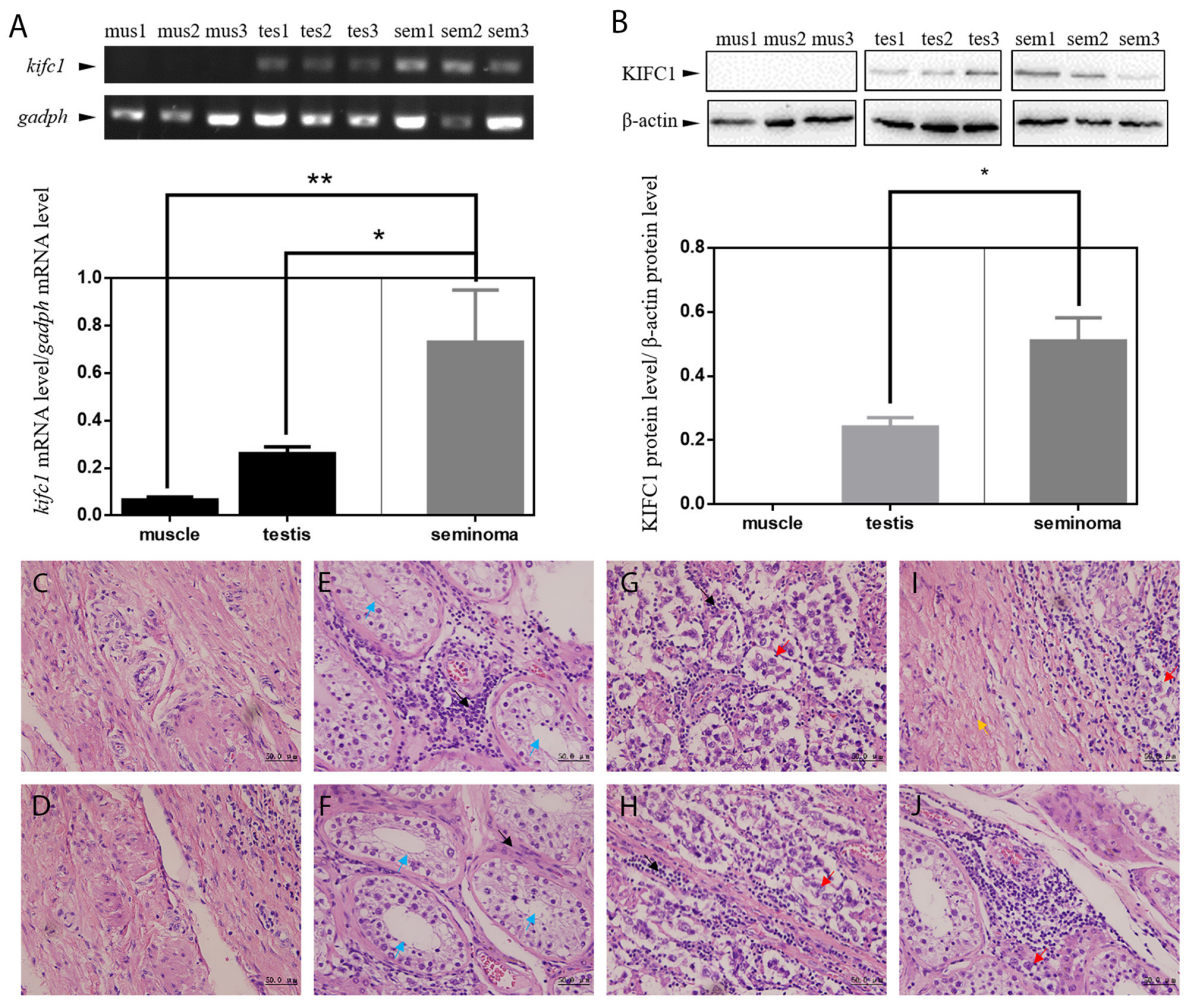
KIFC1 is enriched in human seminoma samples in both mRNA and protein level **(A)** Comparison of mRNA expression level among human muscle (mus), testis (tes) and seminoma (sem) tissue samples. *kifc1* mRNA is enriched in seminoma samples. **(B)** Comparison of protein expression level among human muscle (mus), testis (tes) and seminoma (sem) tissue samples. KIFC1 protein is enriched in both testis samples and seminoma samples, and KIFC1 protein expression level of seminoma samples is significantly higher than that of testis samples. **(C-J)** HE staining results of human muscle tissues (**C, D**) and testis tissues (**E, F**) near the seminoma tissue and the seminoma tissue (**G, H**) from seminoma patient. (**I**) The transition of the seminoma to the muscle tissue. (**J**) The invasion of the cancer cells into the testis tissue. Black arrows show infiltrating lymphocytes. Blue arrows show seminiferous tubules of normal testis tissue. Red arrows show cancer cells. Yellow arrow shows muscle tissue. In the both testis and seminoma, a number of lymphocyte infiltration with bleeding are showed. In seminoma tissues, compared with normal tissues, seminiferous tubules were disrupted and replaced by seminoma cells with loose nuclei and watery cytoplasm.

Pannu et al. performed an *in silico* research to examine KIFC1 protein level of various of human tumors and found that KIFC1 was significantly enriched in lung, breast, glioblastoma, colon and cervical tumors compared to their corresponding normal tissues [20]. However, there are no data relating to testis cancer, which is the most common cancer among young men. Gene expression profiles of normal testis tissue are also not complete on database such as GEO (Gene Expression Omnibus), TCGA and ICGA. So the first thing we did was to evaluate and compare the KIFC1 expression level in muscle, testis and seminoma tissue to determine whether KIFC1 is also important in testis cancer development. We first used semi-quantitative RT-PCR to detect endogenous *kifc1* mRNA level in human tissue samples. A 358-bp cDNA fragment of *kifc1* was amplified in muscle, testis and seminoma tissues (Figure 1A). We found that *kifc1* mRNA was highly expressed in both testis (by 2.79 folds) and seminoma tissues (by 11.16 folds, and the expression in seminoma tissues was also significantly higher than that in testis tissue. We then used western blot to quantify the endogenous protein expression level of KIFC1 among human muscle, testis and seminoma tissues (Figure 1B). There was no detectable KIFC1 expression in muscle tissues, and the KIFC1 protein level in seminoma tissues was also significantly higher than that in testis tissues.

The high expression of KIFC1 in seminoma tissue indicates its possible importance, so we continued to explore the localization of KIFC1 of both seminoma and testis tissue samples. HE staining of human muscle (Figure 1C, 1D) and testis (Figure 1E, 1F) near the seminoma tissue and the seminoma tissue (Figure 1G, 1H) show different histological properties of different tissue types. The unique structure of testis convoluted tubule disappears in the seminoma. Compared with muscle tissue and testis tissue, the cells are round and of even sizes. The nuclei are large and at the middle of the cell (Figure 1C-1H). Figure 1I shows the transition of the seminoma to the muscle, Figure 1J shows the invasion of the cancer cells into the testis.

**Figure 2:**
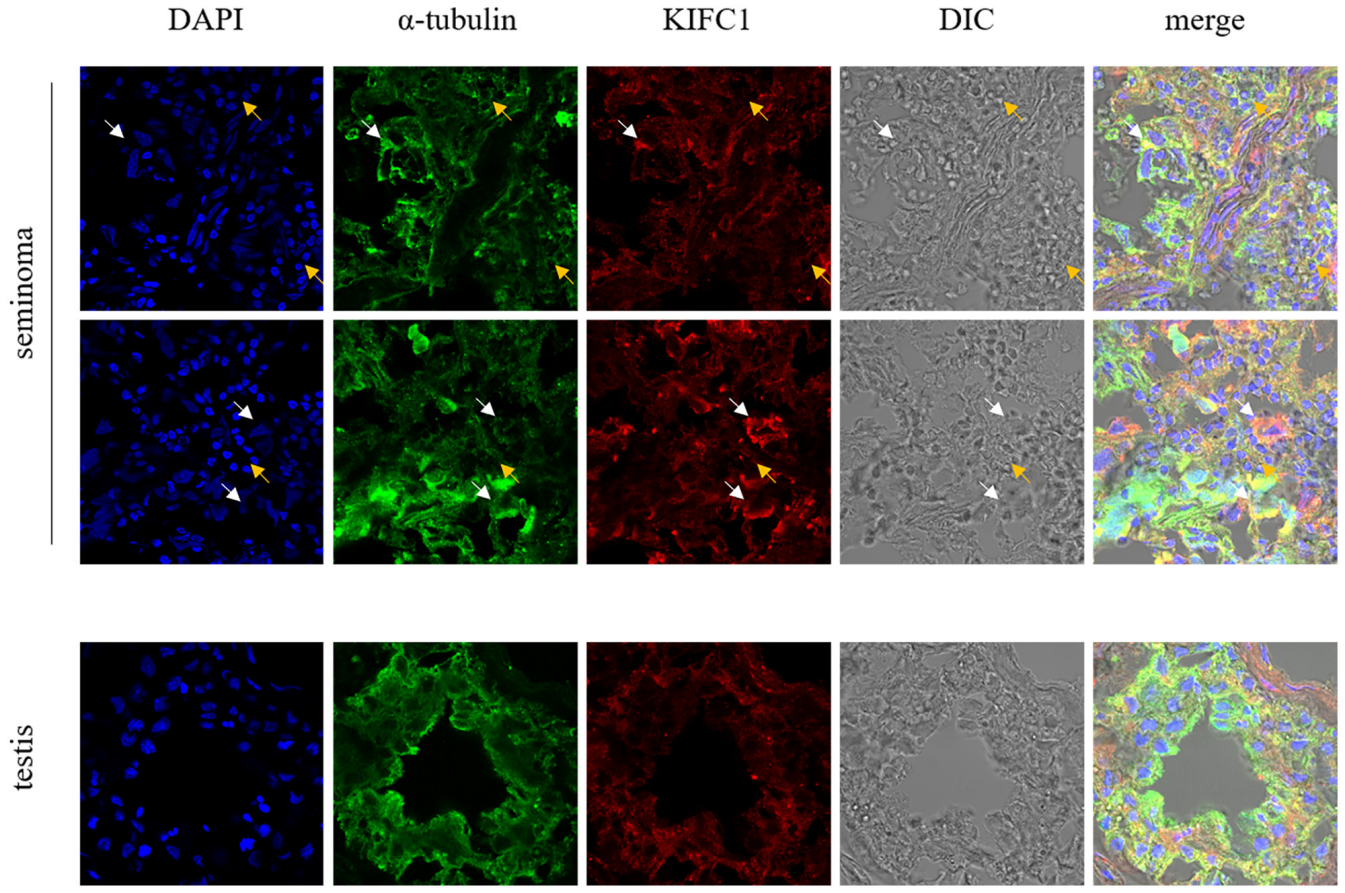
Localization of KIFC1 in seminoma tissue samples and nearby testis tissue samples from human determined by immuno-florescent staining KIFC1 is omnipresent in both seminoma samples and testis samples and co-localizes with MT. White arrows show seminoma cells with loose nuclei and watery cytoplasm, in which KIFC1 signal is strong. Yellow arrows show other cells such as lymphocytes, in which KIFC1 signal is weak. Compared with normal testis tissue, in seminoma tissues the characteristic structure of seminiferous tubules is completely disrupted, and KIFC1 has a tendency to be concentrated around the loose nuclei.

Figure 2 shows the distribution of KIFC1 in different human tissues. In both testis and seminoma tissues, KIFC1 and tubulin show obvious co-localization. This indicates that KIFC1 mainly functions on MT. While KIFC1 is omnipresent in all kinds of cells, in testis tissues, KIFC1 signal mainly concentrated in spermatocytes, which corresponds to previous findings of our lab in researches of KIFC1 in invertebrates [38, 39]. Specifically, in seminoma tissue, KIFC1 signal tends to be concentrated around the nucleus with less intense DNA. This indicates that KIFC1 may possess a unique role during cell cycle

### Loss-of-function of KIFC1 in cancer cells

**Figure 3:**
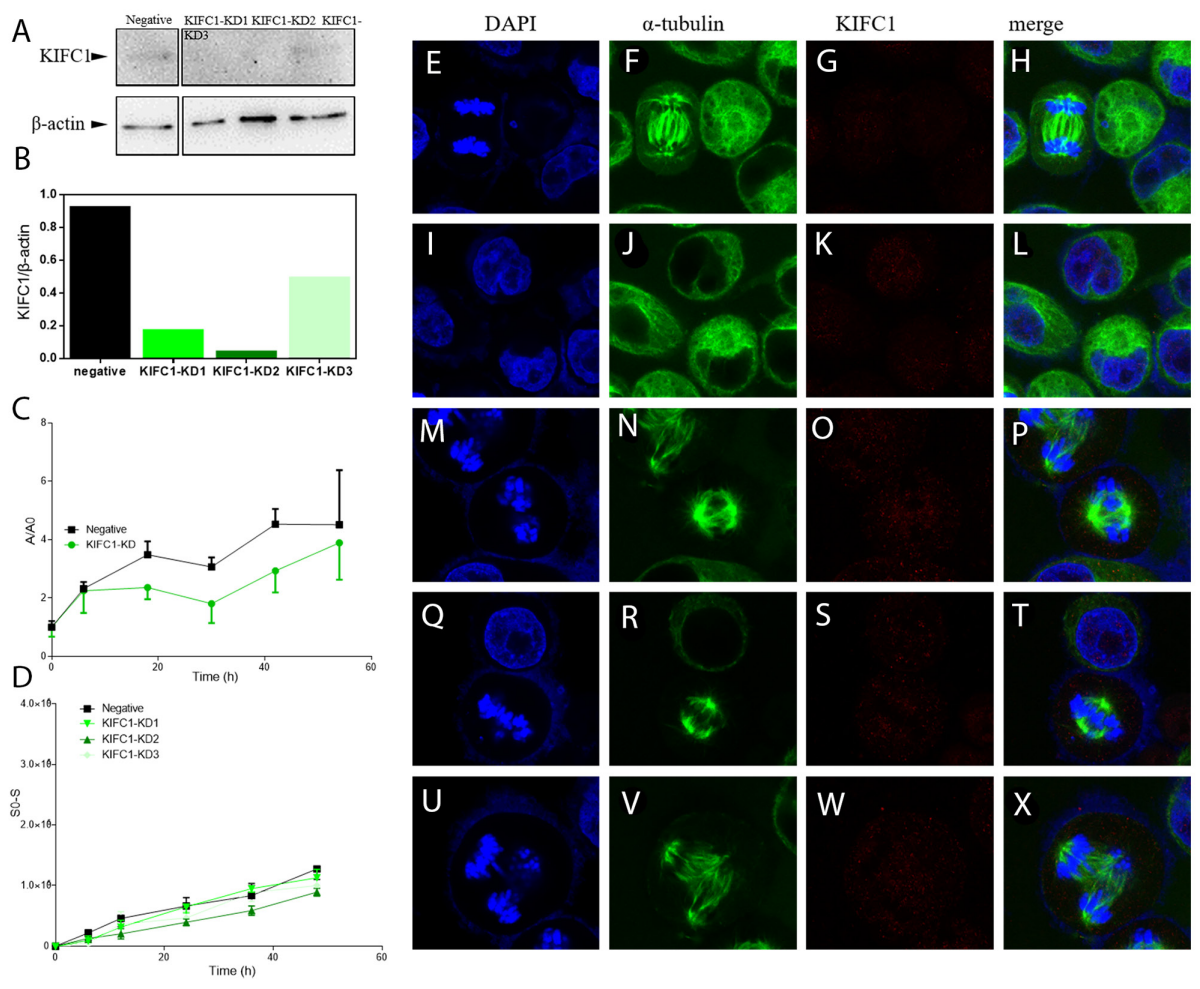
Knock down of KIFC1 in HeLa cells by RNAi **(A)** WB analysis of knock down efficiencies using three different sets of siRNA. **(B)** Localization of KIFC1 in HeLa cells determined by immuno-florescent staining. **(C)** Quantification of WB band intensity from (**A**). The knock down efficiency is as follows: KD2 > KD1 > KD3. **(D)** Growth curve of cells with KIFC1 knock down. The negative control is transfected with siRNA targeting GFP. A=A450-A650, and A0 represents the A value at the time point of 0. **(E-X)** Dynamic behavior of cells with different levels of KIFC1 knock down was determined by wound-healing assay.

To further illustrate KIFC1’s role in cancer cells, we continued to explore the loss-of-function of KIFC1 while cell division. We designed and synthesized 3 different siRNAs to knock down KIFC1 in cells (Table 2). Western blot results show that we can successfully knock down KIFC1 in HeLa cells, and the knocking down efficiency is of gradient (Figure 3A). The knocking down efficiency is in the following order: KIFC1-KD2>KIFC1-KD1>KIFC1-KD3 (Figure 3B). After the knock down of KIFC1, we observe several abnormalities of the cells. It seems that in the prophase the cells are having difficulty in forming only two MTOC (microtubule organizing center) (Figure 3E-3H, 3I-3L). The occurrence of multi-pole spindle is significantly increased (Figure 3M-3P, 3Q-3T, 3U-3X), which corresponded to findings from former researches [40]. And the lining up of the chromosomes in metaphase is also disrupted (Figure 3M-3P, 3Q-3T). The occurrence of anaphase cells is significantly increased, indicating that the anaphase is prolonged (Figure 3E-3H). We next examine the influence of KIFC1 on proliferation ability of the cells. We found that the proliferation rate of the cells with KIFC1 knocked down is decreased in total (P<0.05) (Figure 3C). The curve KIFC1-KD is an intergradation of the data from KIFC1-KD1, KIFC1-KD2 and KIFC1-KD3. As for KIFC1 dose response on the migration ability of the cells, contrary to what happened in NSCL [12], it seems that KIFC1 does not have a significant influence on the migration rate of the HeLa cells (Figure 3D).

### Localization of KIFC1 during cell cycle

**Figure 4:**
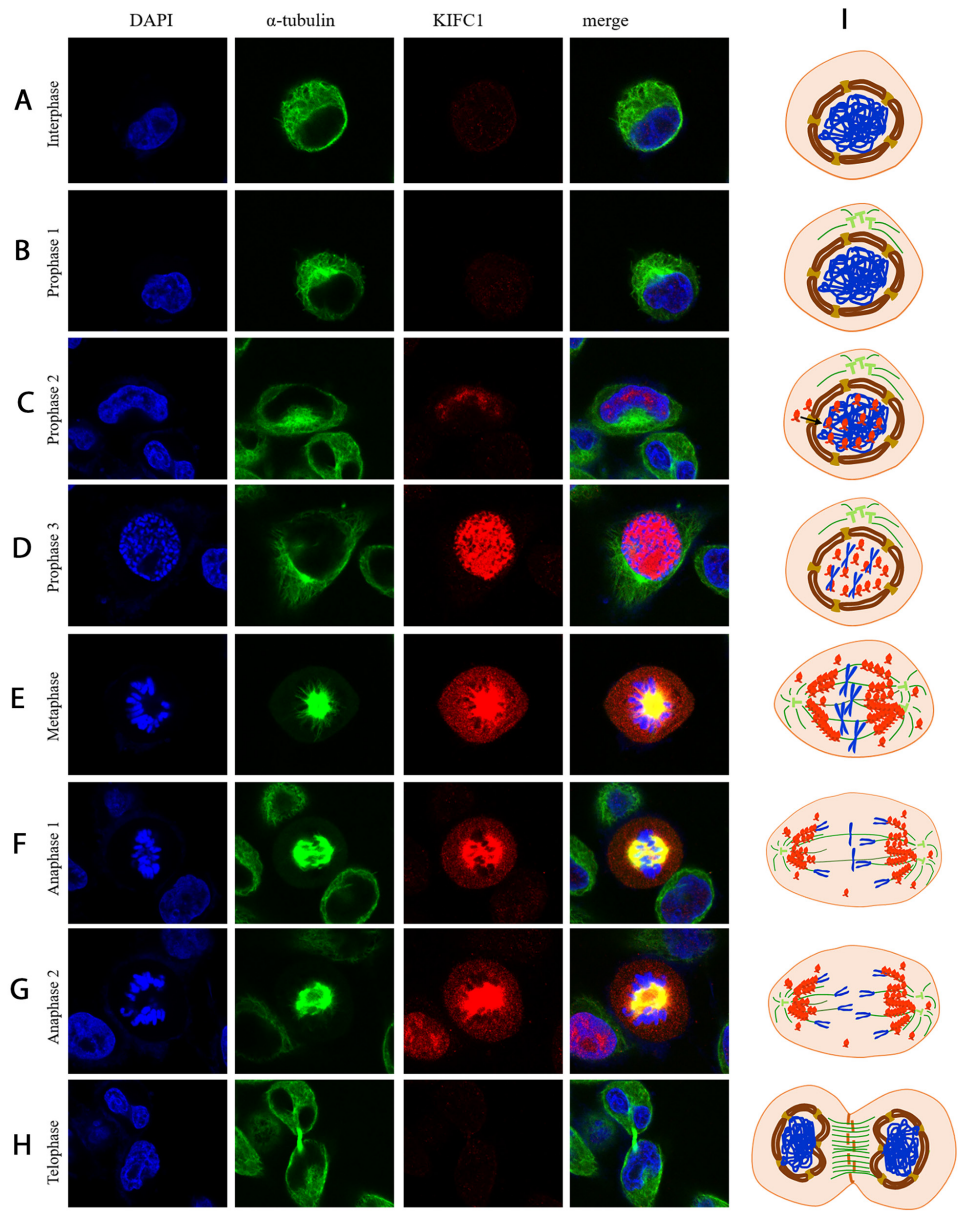
Localization of KIFC1 during cell cycle in HeLa cells determined by immuno-florescent staining **(A-H)** KIFC1 is rapidly transported into and concentrated in the nucleus upon synthesized. In metaphase, KIFC1 is mainly localized on the spindle, but some of it also spread in the cytoplasm. Later on, KIFC1 is degraded after mitotic exit. **(I)** A model illustrating the localization of KIFC1 during the cell cycle.

To this point, it’s fair to say that KIFC1 is able to facilitate proper cell division of cancer cells, so our next step was to explore the action of KIFC1 during cell cycle so as to find out how KIFC1 functions. So we next examined the localization of endogenous KIFC1 during cell cycle by immune-florescent staining. During the interphase and early prophase, KIFC1 protein level is barely detectable (Figure 4A, 4B). The KIFC1 signal then becomes intense and immediately transported into the nucleus immediately after it is synthesized along the progression of the prophase (Figure 4C, 4D). KIFC1 is significantly co-localized with the spindle during the metaphase and the anaphase, but there are also free KIFC1 molecules wandering in the cytoplasm (Figure 4E-4G). Finally, KIFC1 signal vanishes in the telophase (Figure 4H).

### Function analysis of KIFC1 motor domain/ Expression of ΔC-pCMV-N-FLAG plasmid

**Figure 5:**
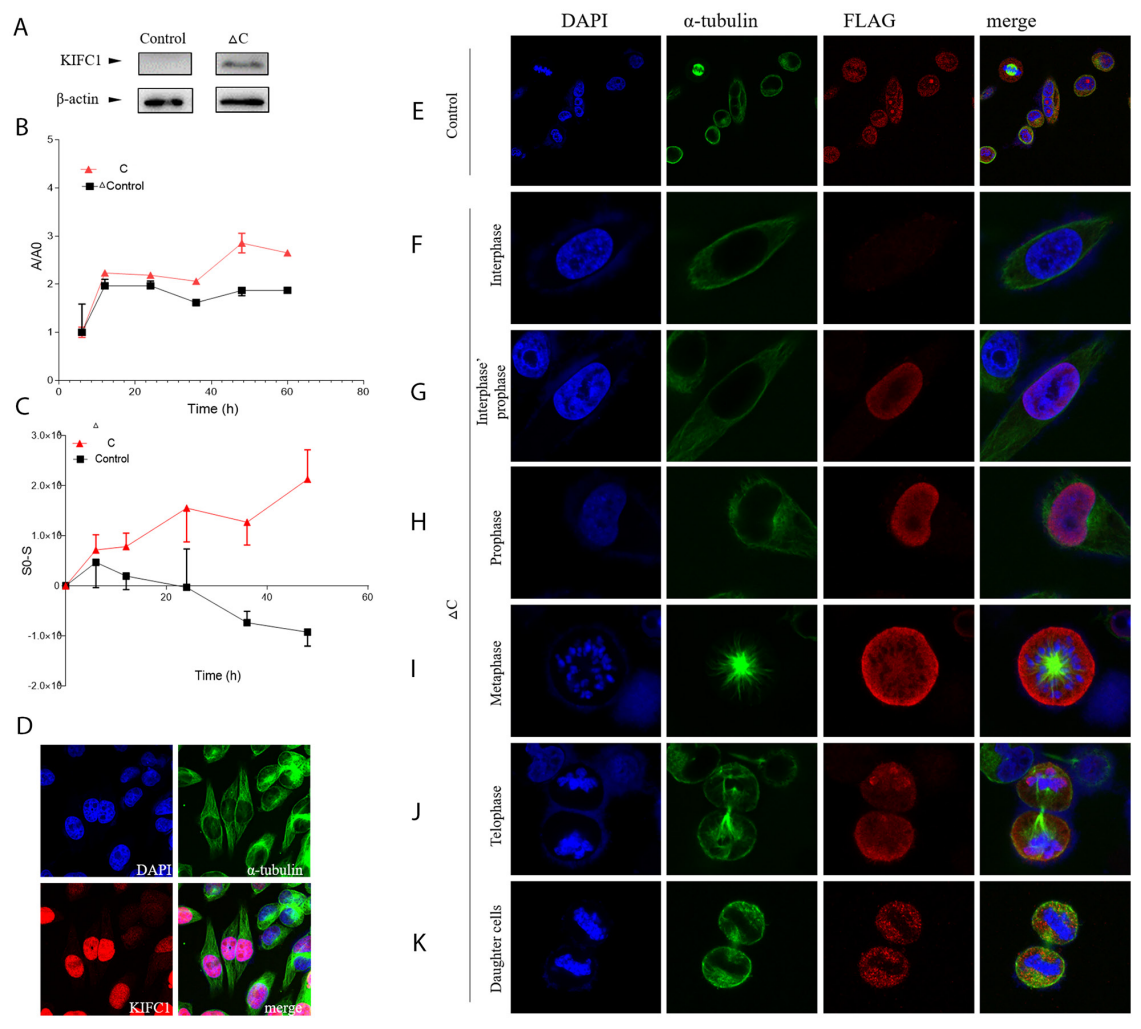
FLAG-tagged ΔC in HeLa cells **(A)** FLAG-tagged ΔC can be expressed in HeLa cells. **(B)** Localization of pCMV-N-FLAG plasmid (control) and FLAG-tagged ΔC during cell cycle in HeLa cells determined by immuno-florescent staining. **(C)** Growth curve of cells transfected with FLAG-tagged ΔC or transfected pCMV-N-FLAG plasmid (control). **(D)** Dynamic behavior of cells transfected with FLAG-tagged ΔC or transfected pCMV-N-FLAG plasmid (control) determined by wound-healing assay. **(E-K)** Endogenous KIFC1 will be entering the nucleus and finally be concentrated in the nucleus during prophase.

To further explore the function of each domain of KIFC1, we constructed and successfully expressed the ΔC-pCMV-N-FLAG plasmid in the cells (Figure 5A). This plasmid expresses a FLAG-tagged KIFC1 with motor domain truncated. And this domain is also known as the non-catalytic MT binding domain. The control FLAG signal in immune-florescent staining result showed that the FLAG peptide itself distributes in the cell evenly in different stages during cell cycle, whereas the ΔC-FLAG peptide does not (Figure 5E-5K). ΔC-FLAG peptide starts to be expressed in interphase and then immediately be transported into the nucleus just like what endogenous KIFC1 is (Figure 5D, 5F, 5G). However, ΔC-FLAG peptide fails to be co-localized with the spindle during metaphase as well as telophase (Figure 5I, 5J). It continues to fail to be degraded in the telophase, and even continues to exist in daughter cells (Figure 5J, 5K).

We next examined the proliferation ability and the dynamic behavior of cells with expressed ΔC-FLAG peptide. Paired t test showed that the proliferation rate of cells with expressed ΔC-FLAG peptide is significantly higher than control group (p<0.05), and the migration rate determined by wound-healing assay of cells with expressed ΔC-FLAG peptide is also significantly higher than control group (p<0.05).

## DISCUSSION

### KIFC1 may be essential in seminoma cell division

We are the first to test and find out that KIFC1 is significantly enriched in seminoma tissue samples in both mRNA level (Figure 1A) and protein level (Figure 1B). The correspondence of the increasing expression level of mRNA and protein in different tissue samples indicate that *kifc1* expression might be regulated and elevated mainly at transcription level in seminoma cells. At the meantime, we still cannot rule out the possibility that KIFC1 can also be up-regulated at the translation level. An interesting explanation of the enrichment of KIFC1 in cancer cells is that *kifc1* gene is observed to be amplified in HeLa cells [20], so the amplified *kifc1* gene might also amplify the mRNA transcribed. However, how KIFC1 is up-regulated still need further illustration.

KIFC1 is vastly distributed in both seminoma and testis tissue samples (Figure 2). It is not surprising to find KIFC1 in human testis samples, as it is long found that KIFC1 is essential in acrosome formation in mouse testis with its vesicle trafficking activity [28]. However, according to HE staining of seminoma and testis tissue samples, the unique structures of testis are totally disappeared in seminoma tissues (Figure 1C-1J), which means that the KIFC1 should have been used for acrosome formation in mammalian testis is no longer used for the same purpose in seminoma tissue, instead, the amount of KIFC1 in seminoma tissue is not decreased. This further indicates that KIFC1 must have unique role in seminoma cells. Immuno-florescent result further indicate that KIFC1 tends to concentrate around less condense nuclei, which normally represents for active cell division. To this end, we conclude that KIFC1 might be essential in seminoma cell division.

### KIFC1 may be essential in cell division, but not essential in metastasis in HeLa cells

The loss-of-function of KIFC1 in cancer cells renders a lowered proliferation rate (Figure 3C), indicating that KIFC1 might be essential in proper cancer cell division. From previous studies, the knock down of KIFC1 can increase the ratio of multi-pole spindles as shown in Figure 3E-3X [40], and the overexpression of KIFC1 should increase the ratio of mono-polar spindles [37]. However, mono-polar spindles are hard to detect in both cells with ΔC-pCMV-N-FLAG and cells with pCMV-N-FLAG negative control. This corresponds to the fact that the motor domain of KIFC1 is essential for its crosslinking and gliding on MT [33]. Given the fact that the overexpression of ΔC increases the cell proliferation (Figure 5C), the way KIFC1 shortens the cell division cycle may not limited to its function on spindle [20]. KIFC1’s activity on MT beside spindle and/or its interaction with cell cycle proteins may also contribute.

During the cell cycle, KIFC1 may also contribute to the lining up of the chromosomes on the metaphase plate as the placing of chromosomes on metaphase plate is observed to be disrupted with KIFC1 knock down (Figure 3E-3X). This deduction is supported by the fact that the depletion of KIFC1 can rescue the poleward movement of chromosomes in cells depleted of B56 family proteins [41]. Besides, APC/C^CDH1^ (anaphase promoting complex/cyclosome), an E3 ligase which interacts with KIFC1 [37], is thought to regulate chromosome segregation [42] probably by facilitating degradation of securin, cyclin B [43] and separase inhibitors [20, 42, 44, 45]. The fact that KIFC1 is also modulated by anaphase promoting complex APC/C^CDH1^ indicates KIFC1 may also have roles beyond MT organization. Our result that the anaphase is prolonged after the knock down of KIFC1 (Figure 3E-3X) also supports this deduction.

KIFC1 is reported to be essential in metastasis of both NSCL as well as triple-negative breast cancer [12, 23]. However, according to our wound-healing assay results for cells with different levels of KIFC1 knocked down (Figure 3D), the knock down of KIFC1 seems to be non-essential for cell migration.

### Localization and function of KIFC1 during cancer cell cycle

Based on immuno-florescent staining results (Figure 4A-4H), we are able to propose a model of activity of KIFC1 during cancer cell cycle (Figure 4I).

KIFC1 becomes detectable during prophase. In the prophase, KIFC1 is perfectly co-localized with DAPI. This indicates two conclusions. For one thing, KIFC1 can be exported into the nucleus during the prophase. While the reason why KIFC1 has to be in the nucleus remains unknown, our speculation is that this might relate to bare double-stranded DNA transportation activity of KIFC1 [46]. Our result also shows that ΔC-pCMV-N-FLAG can still be transported into the nucleus during the prophase (Figure 4). These correspond to previous study that mammalian KIFC1 is associated with importin β, which is known for transporting proteins into the nucleus by binding to nuclear localization sequences (NLS), in mouse testis [28]. And also the interaction sequence NLS on KIFC1 with importin β is identified to localize on the tail domain [33, 47]. For another, KIFC1 might be exported into the nucleus right after it is synthesized. In metaphase and anaphase, KIFC1 co-localizes with the spindle, indicates that the main function of KIFC1 is on microtubule. This correspond to the indicated main function of KIFC1, that KIFC1 crosslinks the MT [33–36]. However, besides concentrated on the microtubule, KIFC1 is also observed to be distributed in cytoplasm. This indicates that the binding between KIFC1 and MT might be dynamic. This speculation is further supported by our result that ΔC-FLAG peptides can no longer co-localize with the spindle (Figure 5E-5K). The distribution of KIFC1 in cytoplasm can also indicate that KIFC1 might bind to MT that does not participate in spindle formation as well. Our speculation is that KIFC1 might transport organelles such as ER, vesicles and even mitochondria. This speculation is also supported by the fact that KIFC1 is recruited during trafficking of early endocytic vesicles in human liver cells [48, 49]. In telophase and after the mitotic exits, KIFC1 accomplishes its duty and is subsequently degraded.

## CONCLUSIONS

Our work here indicates that KIFC1 participates in facilitating proper division of seminoma cancer cells. KIFC1 may be essential for cancer cell division but may not be essential for cell migration of HeLa cells. The functions of KIFC1 during cell division are beyond MT organization. KIFC1 might also be essential for the lining up of the chromosomes on the metaphase plate, the trafficking of organelles during cell cycle, and KIFC1 can also regulate the length of the anaphase and mitotic exit. The tail domain of KIFC1 might be essential for the dynamic disassociation of KIFC1 and KIFC1’s entrance into the nucleus, and the motor domain of KIFC1 might be essential for the degradation of KIFC1.

## MATERIALS AND METHODS

### Human tissue and cell culture

Human seminoma tissues, testis tissues and muscle tissues were obtained after the “Patient Consent Form” were signed and documented by the First Hospital of Zhejiang Province (The First Affiliated Hospital of Zhejiang University) under a protocol issued to Dr. Fu-Qing Tan. These samples were obtained from surgeries for treating seminoma from one patient. Fresh samples were identified and dissected on ice and conserved in either iced PBS (phosphate buffer saline solution, pH 7.4) or iced PFA (PBS with 4% paraformaldehyde). Some samples kept in PBS were immediately used for RNA extraction, the rest of the samples kept in PBS were kept at -20 °C for protein extraction, and the samples kept in PFA were immediately used for frozen section.

We cultured HeLa cells with Gibico^®^ DMEM supplemented with 10% of Gibico^®^ FBS and penicillin/streptomycin at 37°C with 5% CO_2_ incubation.

### RNA extraction and reverse transcription

Total RNA samples were prepared from human tissue samples including muscle, testis and seminoma lysed by RIPA Lysis Buffer (Beyotime). The lysates were then dissolved in RNAiso Plus (TaKaRa BIO INC), and then extracted with chloroform (Sinopharm Chemical Reagent Co., Ltd). Isopropyl alcohol (Zhejiang Hangzhou Shuanglin Chemical Reagent Factory) was then added to the supernatant to precipitate the RNA. The RNA pellets were then washed with cold 100% and 75% ethanol (Sinopharm Chemical Reagent Co., Ltd). Pure RNA pellets are dissolved in RNase free water and stored at -40°C. The reverse transcription was conducted with PrimerScript™ RT Kit (TaKaRa BIO INC.) to establish cDNA profiles of each tissue samples.

Two specific primers of KIFC1 were designed by NCBI BLAST Primers to detect the endogenous amount of *kifc1* mRNA (Table 1A, 1B). Two specific primers of GADPH were designed as the loading control (Table 1C, 1D). Three different tissues (muscle, testis, seminoma) and three samples of each were used for this assay. The PCR procedures were as follows: 94°C for 5 min; 34 cycles of 94°C for 30s, 55°C for 30s, 72°C for 30s; 72°C for 10min. The PCR products were detected by agar gel electrophoresis. The results were analyzed with ImageJ and GraphPad Prism 6 software.

**Table 1:** Primers used in the study

Primer name		Sequence	Purpose
A	KIFC1XYX-F2	AGAAACCCAGCAAACGTCCA	Semi-quantitative RT-PCR for endogenous *kifc1*
B	KIFC1XYX-R2	AGTTGGGACATCAGTCCCCT	Semi-quantitative RT-PCR for endogenous *kifc1*
C	GADPH-F	ACCACAGTCCATGCCATCAC	Internal control of semi-quantitative RT-PCR
D	GADPH-R	TCCACCACCCTGTTGCTGTA	Internal control of semi-quantitative RT-PCR
E	KIFC1KC-XYX-F1	CCGCTCGAGATGGATCCGCAGAGGTCCCC	Construction of ΔC-pCMV-N-FLAG plasmid
F	KIFC1KC-XYX-B1	TGCTCTAGAGCCCTTGAGTTCCTGCAGCTG	Construction of ΔC-pCMV-N-FLAG plasmid
G	M13F	CGCCAGGGTTTTCCCAGTCACGAC	Colony PCR when constructing the Δ C-pCMV-N-FLAG plasmid
H	M13R	AGCGGATAACAATTTCACACAGGA	Colony PCR when constructing the Δ C-pCMV-N-FLAG plasmid

### Protein extraction

The tissues temperately conserved in PBS on ice were lysed in a RIPA Lysis Buffer (Beyotime) with proteinase inhibitors on ice for 20 min using homogenizer workcenter (IKA^®^ T10 basic). Then we spanned the lysate at 14,000g for 10 min at 4°C, collected the supernatant, added sample buffer, and boiled at 95°C for 7min. The samples were stored at -40°C and were now ready to be subjected to SDS-PAGE.

### Western blot

The samples were run in 5% of spacer gel and 10% of separation gel. The proteins were then transferred to a PVDF membrane (Immobilon^®^-P). The blot was blocked in 5% skimmed milk in PBST (PBS with 1%BSA and 0.05% Tween 20) for 1 h, and then incubated in specific primary antibodies with recommended dilution at 4°C overnight. Washed the blot again with PBST and incubated with secondary antibodies with recommended dilution at room temperature for 1 hour. Developed the blot with eECL Western Blot Kit (CWBio) and imaged them with Tanon-5200 (Shanghai sky Technology Co., Ltd.). Monoclonal Rabit anti-KIFC1 antibody was purchased from Abcam and was used 1:1000. The Mouse polyclonal anti-β-actin antibody was obtained from BBI Lifescience and was used 1:2000. The FLAG Tag Rabbit Polyclonal Antibody was obtained from Beyontime and was used 1:750. The HRP-labeled Goat Anti-Rabbit IgG (H+L) and HRP-labeled Goat Anti-Mouse IgG(H+L) were both obtained from Beyotime and were used 1:2000.

### RNA interfering

#### Preparation of siRNAs

We used TaKaRa *in vitro* Transcription T7 Kit to synthesize the siRNAs of interest. Three different sets of oligos were designed for knocking down of endogenous KIFC1, and one set of oligos were designed for negative control (Table 2). The negative control oligos are targeting GFP, which is not existed in human. These oligos were designed and the siRNAs of interest were synthesized following TaKaRa *in vitro* Transcription T7 Kit protocol.

**Table 2:** Oligos used for RNAi in the study

Oligo name		Sequence
KIFC1-KD1	KIFC1si1-1	GATCACTAATACGACTCACTATAGGGCAAGCTACGTAGAGATCTATT
	KIFC1si1-2	AATAGATCTCTACGTAGCTTGCCCTATAGTGAGTCGTATTAGTGATC
	KIFC1si1-3	AACAAGCTACGTAGAGATCTACCCTATAGTGAGTCGTATTAGTGATC
	KIFC1si1-4	GATCACTAATACGACTCACTATAGGGTAGATCTCTACGTAGCTTGTT
KIFC1-KD2	KIFC1si2-1	GATCACTAATACGACTCACTATAGGGCCTCAACTCTCTACGCTTTTT
	KIFC1si2-2	AAAAAGCGTAGAGAGTTGAGGCCCTATAGTGAGTCGTATTAGTGATC
	KIFC1si2-3	AACCTCAACTCTCTACGCTTTCCCTATAGTGAGTCGTATTAGTGATC
	KIFC1si2-4	GATCACTAATACGACTCACTATAGGGAAAGCGTAGAGAGTTGAGGTT
KIFC1-KD3	KIFC1si3-1	GATCACTAATACGACTCACTATAGGGGGACTTAAAGGGTCAGTTATT
	KIFC1si3-2	AATAACTGACCCTTTAAGTCCCCCTATAGTGAGTCGTATTAGTGATC
	KIFC1si3-3	AAGGACTTAAAGGGTCAGTTACCCTATAGTGAGTCGTATTAGTGATC
	KIFC1si3-4	GATCACTAATACGACTCACTATAGGGTAACTGACCCTTTAAGTCCTT
Negative	GFPsi-1	GATCACTAATACGACTCACTATAGGGGGGATGTCTCACATCTTGTTT
	GFPsi-2	AAACAAGATGTGAGACATCCCCCCTATAGTGAGTCGTATTAGTGATC
	GFPsi-3	AAGGGATGTCTCACATCTTGTCCCTATAGTGAGTCGTATTAGTGATC
	GFPsi-4	GATCACTAATACGACTCACTATAGGGACAAGATGTGAGACATCCCTT

#### Transfection

We used Invitrogen^™^ Lipofectamine^®^ 3000 Kit to transfect synthesized siRNA into 24-well plate tissue cultures. For each well of cells, 0.75μl of Lipo3000 was diluted in 25μl of Gibico^®^ Opti-MEM; 500ng of siRNA were diluted in 25μl of Gibico^®^ Opti-MEM. We mixed the diluted Lipo3000 and diluted siRNA well and incubated for 5min. We added the mixed liquid into one well of cells (confluence 70%-90%), incubated the cells in 37°C with 5% CO_2_ for 24 hours and then used the cells for further analysis.

### Overexpression of recombinant plasmids in cells

#### Construction of recombinant plasmids

One set of primers (Table 1E, 1F) were used to amplify the *kifc1* gene with truncated motor domain (ΔC) from testis cDNA profile with PrimerSTAR^®^ HS DNA Polymerase (TaKaRa). The PCR procedures were as follows: 34 cycles of 98°C 15s and then 68°C 2min. The PCR product was then tested on the agrose gel and purified with SanPrep column PCR Purification Kit (Sangon Biotech). Digest the PCR product and the pCMV-N-FLAG with final concentration of 0.75U/μl restriction endonucleases (each): XhoI and XbaI (TaKaRa). We used TaKaRa T4 ligase Kit for ligation overnight at 18°C and transformed the ligation reaction into DH5α. We used one set of primers that are priming at sequence on the pCMV-N-FLAG backbone (Table 1G, 1H) to do the colony PCR for screening, and sequencing was performed by Biosune. We then use EZNA Plasmid Miniprep Kit II (Omega) to prepare endotoxin-free plasmids.

#### Transfection

We used Invitrogen™ Lipofectamine^®^ 3000 Kit to transfect the ΔC-pCMV-N-FLAG plasmid into 24-well plate tissue cultures. Transfected pCMV-N-FLAG plasmid was used as a control.

### Immuno-fluorescent staining

#### Frozen section and tissue slides preparation

Three different tissues (muscle, testis and seminoma) were first cut into 8mm^3^ pieces, then fixed in PFA for 4h at 4°C, and then dehydrated in 0.5M sucrose solution for 4-8h (renew the total volume of 0.5M sucrose solution once). The fixed and dehydrated tissue pieces were then embedded in OCT (opti-mum cutting temperature compound) compound and stored in -40°C. The samples were sectioned to 5μm and mounted on polylysine-coated slides. The prepared samples were stored at -40°C.

#### Cell slides preparation

100ul glutin were added into each well of 24-well plate and incubate for 30min at 37°C with 5% CO_2_, and were then removed. We then plated one slide of round cover glass slide into each well and seeded the cells. The cells slides that were ready for immune-florescent staining were fixed with 4% PFA for 15min and then we washed the slides with cold PBS. The cells on the slides were permeablized with 0.25% TritonX-100/PBS for 10min and then were washed again with cold PBS. The prepared samples were used for immune-florescent staining immediately.

#### Immuno-florescent staining

We blocked the slides with 1% BSA/PBST for 30min and then the slides were incubated in primary antibodies overnight at 4°C. The primary antibodies used for labeling KIFC1 and FLAG are the same as the ones used for WB but used in the concentration of 1:200. Wash the slides again with PBST and incubate with secondary antibody. To label KIFC1 or FLAG, we used Alexa Fluor 555-labeled Donkey Anti-Rabbit IgG (H+L) obtained from Beyotime (1:200). To label β-tubulin, we used 488-labeled Monoclonal Anti-beta-Tubulin-FITC antibody produced in mouse obtained from Sigma (1:100). After washed away the secondary, we incubated the slides with DAPI (Beyotime) for 5min. The slides were then washed with PBS and 2 drops of Antifade Mounting Medium (Beyotime) were added. We sealed the slides with nail polish and observed them under the Confocal Laser-scanning Microscope (CLSM510; Carl Zeiss Germany).

### Cell counting

We used Cell Counting Kit-8 (Beyotime) to draw the growth curves of the treated cells. We seeded 1000-2000 cells (with siRNA or plasmid transfected) with 200μl culture media into each well of 96-well plate. The cells were cultured for 6h and then let them attach to the plates. Then we cultured the cells for another 0h, 6h, 18h, 30h, 42h and 54h (5 replicates for each group), and added 10μl of CCK-8 solution from the kit, incubated for 30min at 37°C with 5% CO_2_, and used synergy™ H1Multi-Mode Reader to perform DWSP (double-wavelength spectrophotometry), detecting the OD value under the 450nm as well as the A650 wavelength. The ultimate value A=A450-A650.

### Wound-healing assay

We cultured the treated cells in 12-well plate until they reached 100% confluence. Then we used a 200μl pipette tip (yellow) to make a straight scratch. The cells were culture for 0h, 6h, 12h, 24h, 36h and 48h (3 replicates for each group). We observed the cells under the Phase-Contrast Inverted microscope (Olympus) and measured the area of the gap using ImageJ. The migration rate of the cells ∝ A0-A.

## References

[1] Siegel RL, Miller KD, Jemal A (2016). Cancer statistics, 2016. CA Cancer J Clin.

[2] Kuwana T, Erlander M, Peterson PA, Karlsson L (1996). Cloning and expression of HSET, a kinesin-related motor protein encoded in MHC class II region. Mol Biol Cell.

[3] De S, Cipriano R, Jackson MW, Stark GR (2009). Overexpression of kinesins mediates docetaxel resistance in breast cancer cells. Cancer Res.

[4] Kwon M, Godinho SA, Chandhok NS, Ganem NJ, Azioune A, Thery M, Pellman D (2008). Mechanisms to suppress multipolar divisions in cancer cells with extra centrosomes. Genes Dev.

[5] Rath O, Kozielski F (2012). Kinesins and cancer. Nat Rev Cancer.

[6] Xiao YX, Yang WX (2016). KIFC1: a promising chemotherapy target for cancer treatment?. Oncotarget.

[7] Ogden A, Rida PC, Aneja R (2012). Let’s huddle to prevent a muddle: centrosome declustering as an attractive anticancer strategy. Cell Death Differ.

[8] Aneja R, Mittal K, Pannu V, Klimov S, Cantuaria GH, Sams R, Rida CG (2015). A centrosome clustering protein, HSET, as a potential biomarker for ovarian adenocarcinomas. J Clin Oncol.

[9] Ashworth A, Bernards R (2010). Using functional genetics to understand breast cancer biology. Cold Spring Harb Perspect Biol.

[10] Li Y, Lu W, Chen D, Boohaker RJ, Zhai L, Padmalayam I, Wennerberg K, Xu B, Zhang W (2015). KIFC1 is a novel potential therapeutic target for breast cancer. Cancer Biol Ther.

[11] Alekseev B, Vorobyev N, Shegay P, Zabolotneva A, Rusakov I, Buzdin A, Gaifullin N (2014). Identification of novel gene expression markers for bladder cancer diagnostics. J Urol.

[12] Grinberg-Rashi H, Ofek E, Perelman M, Skarda J, Yaron P, Hajdúch M, Jacob-Hirsch J, Amariglio N, Krupsky M, Simansky DA, Ram Z, Pfeffer R, Galernter I (2009). The expression of three genes in primary non-small cell lung cancer is associated with metastatic spread to the brain. Clin Cancer Res.

[13] Chan JY (2011). A clinical overview of centrosome amplification in human cancers. Int J Biol Sci.

[14] Zhang C, Chen X, Chen X, Wang X, Ji A, Jiang L, Sang F, Li F (2016). miR-135a acts as a tumor suppressor in gastric cancer in part by targeting KIFC1. Onco Targets Ther.

[15] Wu J, Mikule K, Wang W, Su N, Petteruti P, Gharandaghi F, Code E, Zhu X, Jacques K, Lai Z, Yang B, Lamb ML, Chuaqui C (2013). Discovery and mechanistic study of a small molecule inhibitor for motor protein KIFC1. ACS Chem Biol.

[16] Zhang W, Zhai L, Wang Y, Boohaker RJ, Lu W, Gupta VV, Padmalayam I, Bostwick RJ, White EL, Ross LJ, Maddry J, Ananthan S, Augelli-Szafran CE (2016). Discovery of a novel inhibitor of kinesin-like protein KIFC1. Biochem J.

[17] Watts CA, Richards FM, Bender A, Bond PJ, Korb O, Kern O, Riddick M, Owen P, Myers RM, Raff J, Gergely F, Jodrell DI, Ley SV (2013). Design, synthesis, and biological evaluation of an allosteric inhibitor of HSET that targets cancer cells with supernumerary centrosomes. Chem Biol.

[18] Ogden A, Rida PC, Aneja R (2013). Heading off with the herd: how cancer cells might maneuver supernumerary centrosomes for directional migration. Cancer Metastasis Rev.

[19] Ganem NJ, Godinho SA, Pellman D (2009). A mechanism linking extra centrosomes to chromosomal instability. Nature.

[20] Pannu V, Rida PCG, Ogden A, Turaga RC, Donthamsetty S, Bowen NJ, Rudd K, Gupta MV, Reid MD, Cantuaria G, Walczak CE, Aneja R (2015). HSET overexpression fuels tumor progression via centrosome clustering-independent mechanisms in breast cancer patients. Oncotarget.

[21] Martin SK, Kyprianou N (2015). Exploitation of the androgen receptor to overcome taxane resistance in advanced prostate cancer. Adv Cancer Res.

[22] Zou JX, Duan Z, Wang J, Sokolov A, Xu J, Chen CZ, Li JJ, Chen HW (2014). Kinesin family deregulation coordinated by bromodomain protein ANCCA and histone methyltransferase MLL for breast cancer cell growth, survival, and tamoxifen resistance. Mol Cancer Res.

[23] Ogden A, Oprea-Ilies G, Rida PCG, Nickleach D, Liu Y, Cantuaria G, Aneja R, Nuclear HSET (2014). a predictor for metastasis, disease relapse, and poor survival, is a racial disparity biomarker in triple-negative breast cancer patients. Cancer Epidemiol Biomarkers Prev.

[24] Nath S, Bananis E, Sarkar S, Stockert RJ, Sperry AO, Murray JW, Wolkoff AW (2007). Kif5B and Kifc1 interact and are required for motility and fission of early endocytic vesicles in mouse liver. Mol Biol Cell.

[25] Dutoya S, Gibert S, Lemercier G, Santarelli X, Baltz D, Baltz T, Bakalara N (2001). A novel C-terminal kinesin is essential for maintaining functional acidocalcisomes in Trypanosoma brucei. J Biol Chem.

[26] Mukhopadhyay A, Nieves E, Che F, Wang J, Jin L, Murray JW, Gordon K, Angeletti RH, Wolkoff AW (2011). Proteomic analysis of endocytic vesicles: Rab1a regulates motility of early endocytic vesicles. J Cell Sci.

[27] Mukhopadhyay A, Quiroz JA, Wolkoff AW (2014). Rab1a regulates sorting of early endocytic vesicles. Am J Physiol Gastrointest Liver Physiol.

[28] Yang WX (2003). C-terminal kinesin motor KIFC1 participates in acrosome biogenesis and vesicle transport. Biol Reprod.

[29] Hall VJ, Compton D, Stojkovic P, Nesbitt M, Herbert M, Murdoch A, Stojkovic M (2007). Developmental competence of human *in vitro* aged oocytes as host cells for nuclear transfer. Hum Reprod.

[30] Liu Z, Youngquist RS, Garverick HA, Antoniou E (2009). Molecular mechanisms regulating bovine ovarian follicular selection. Mol Reprod Dev.

[31] Truscott M, Harada R, Vadnais C, Robert F, Nepveu A (2008). p110 CUX1 cooperates with E2F transcription factors in the transcriptional activation of cell cycle-regulated genes. Mol Cell Biol.

[32] Sansregret L, Vadnais C, Livingstone J, Kwiatkowski N, Awan A, Cadieux C, Leduy L, Hallett MT, Nepveu A (2011). Cut homeobox 1 causes chromosomal instability by promoting bipolar division after cytokinesis failure. Proc Natl Acad Sci U S A.

[33] Cai S, Weaver LN, Ems-McClung SC, Walczak CE (2009). Kinesin-14 family proteins HSET/XCTK2 control spindle length by cross-linking and sliding microtubules. Mol Biol Cell.

[34] Loughlin R, Riggs B, Heald R (2008). SnapShot: motor proteins in spindle sssembly. Cell.

[35] Mountain V, Simerly C, Howard L, Ando A, Schatten G, Compton DA (1999). The kinesin-related protein, HSET, opposes the activity of Eg5 and cross-links microtubules in the mammalian mitotic spindle. J Cell Biol.

[36] Jiang C, You Q (2013). Kinesin spindle protein inhibitors in cancer: a patent review (2008-present). Expert Opin Ther Pat.

[37] Singh SA, Winter D, Kirchner M, Chauhan R, Ahmed S, Ozlu N, Tzur A, Steen JA, Steen H (2014). Co-regulation proteomics reveals substrates and mechanisms of APC/C-dependent degradation. EMBO J.

[38] Hou CC, Yang WX (2013). Acroframosome-dependent KIFC1 facilitates acrosome formation during spermatogenesis in the caridean shrimp Exopalaemon modestus. PLoS One.

[39] Tan F, Ma X, Zhu J, Yang W (2013). The expression pattern of the C-terminal kinesin gene kifc1 during the spermatogenesis of Sepiella maindroni. Gene.

[40] Zhu C, Zhao J, Bibikova M, Leverson JD, Bossy-Wetzel E, Fan JB, Abraham RT, Jiang W (2005). Functional analysis of human microtubule-based motor proteins, the kinesins and dyneins, in mitosis/cytokinesis using RNA interference. Mol Biol Cell.

[41] Xu P, Virshup DM, Lee SH (2014). B56-PP2A regulates motor dynamics for mitotic chromosome alignment. J Cell Sci.

[42] Chang L, Barford D (2014). Insights into the anaphase-promoting complex: a molecular machine that regulates mitosis. Curr Opin Struct Biol.

[43] Primorac I, Musacchio A (2013). Panta rhei: the APC/C at steady state. J Cell Biol.

[44] Pines J (2011). Cubism and the cell cycle: the many faces of the APC/C. Nat Rev Mol Cell Biol.

[45] Barford D (2011). Structural insights into anaphase-promoting complex function and mechanism. Philos Trans R Soc Lond B Biol Sci.

[46] Farina F, Pierobon P, Delevoye C, Monnet J, Dingli F, Loew D, Quanz M, Dutreix M, Cappello G (2013). Kinesin KIFC1 actively transports bare double-stranded DNA. Nucleic Acids Res.

[47] Wang R, Sperry AO (2008). Identification of a novel Leucine-rich repeat protein and candidate PPI regulatory subunit expressed in developing spermatids. BMC Cell Biol.

[48] Quiroz J, Mukhopadhyay A, Wolkoff AW (2013). Rab1a regulates endocytic vesicle processing through interaction with Kifc1. FASEB J.

